# PCV cap proteins fused with calreticulin expressed into polymers in *Escherichia coli* with high immunogenicity in mice

**DOI:** 10.1186/s12917-020-02527-9

**Published:** 2020-08-27

**Authors:** Chang Liu, Yunchao Liu, Hua Feng, Baolei Zhao, Yumei Chen, Huimin Huang, Pan Wang, Ruiguang Deng, Gaiping Zhang

**Affiliations:** 1 Key Laboratory of Animal Immunology of the Ministry of Agriculture, Henan Provincial Key Laboratory of Animal Immunology, Henan Academy of Agricultural Sciences, Zhengzhou, 450002 Henan China; 2 College of Animal Science and Veterinary Medicine, Henan Agricultural University, Zhengzhou, 450002 Henan China; 3 School of Life Sciences, Zhengzhou University, Zhengzhou, 450001 China

**Keywords:** Porcine circovirus type 2, CRT-cap fusion protein, *Escherichia coli*, Polymers, Immunogenicity

## Abstract

**Background:**

Porcine circovirus type 2 (PCV2) is the main causative agent of porcine circovirus diseases (PCVDs) which causes huge yearly economic losses in the swine industry. Capsid protein (Cap) is the major structural protein of PCV2 that can induce a protective immune response. Therefore, developing a novel and safe subunit vaccine against PCV2 infection is needed.

**Results:**

In this study, the *Cap* gene was bound to the truncated *calreticulin* (CRT) (120–250 aa/120–308 aa) at the N/C terminal, and then the CRT-Cap fusion genes were expressed in *Escherichia coli (E.coli)*. The size-exclusion chromatography and dynamic light scattering (DLS) data showed that the purified recombinant CRT-Cap fusion protein (rP5F) existed in the form of polymers. Immunization with rP5F stimulated high levels of PCV2 specific antibody and neutralization antibody in mice, which were almost identical to those induced by the commercial subunit and inactivated vaccines. The lymphocyte proliferation and cytokine secretion were also detected in rP5F immunized mice. According to the results of PCV2-challenge experiment, the virus loads significantly decreased in mice immunized with rP5F. The data obtained in the current study revealed that rP5F had the potential to be a subunit vaccine candidate against PCV2 in the future.

**Conclusions:**

We have successfully expressed Cap-CRT fusion proteins in *E.coli* and optimized rP5F could form into immunogenic polymers. Mice immunized with rP5F efficiently induced humoral and part of cellular immune responses and decreased the virus content against PCV2-challenge, which suggested that rF5P could be a potential subunit vaccine candidate.

## Background

Porcine circovirus (PCV) is a circular single-stranded DNA virus belonging to the family *Circoviridae* [1]. There are three major genotypes of PCV, namely PCV1, PCV2, and PCV3. PCV1 is nonpathogenic [2], and PCV2 is associated with several diseases, collectively named as porcine circovirus associated disease (PCVAD), which causes reproductive failure and huge economic losses all over the world [3]. PCV3 is a recently identified circovirus that induces cardiac pathology and multi-systemic inflammation [4]. In April 2019, a new circovirus with a distinct relationship to other circoviruses was found in Hunan Province, China and designated as PCV4 (doi:10.1111/TBED.13446). At present, at least five commercial vaccines have been licensed, including the Circovac® vaccine (Merial), Ingelvac CircoFLEX® (Boehringer Ingelheim), Circumvent® (Intervet/Merck), Porcilis® PCV (Schering-Plough/Merck), as well as Fostera™ PCV (Pfizer Animal Health Inc.) [5]. It has been reported that all the commercial vaccines were able to reduce clinical symptoms and improve reproduction to some extent in PCV2 positive farms, while they failed to eradicate this virus from farms [6, 7]. As PCV2 infection may initiate immunosuppression and also cause subsequent failure of the immune response in pigs [8], thus, a more effective vaccine should be developed to prevent PCV2 infections in swine herds. The BALB/c mouse is one of the animal models, as it has a clear background and frees from external interference, it is the most extensively used in PCV2 inactivated or subunit vaccine researches [9, 10].

The genome of PCV2 consists of two major open reading frames (ORFs): ORF1 and ORF2. ORF1 encodes two viral replication-associated proteins, Rep and Rep’ [11]; ORF2 encodes a capsid protein (Cap), which is the primary immunogenic protein of PCV2. Cap contains critical epitopes for inducing a protective immune response, so it has been used as the target for vaccine development [12]. The Cap protein has been expressed in multiple protein expression systems (e.g., insects, mammalian, yeast, and *E. coli* cells) [13, 14] in vitro, whereas only baculovirus insect expression system generates two commercially available PCV2 vaccines [6]. However, low yield and high cost still exist for large scale preparation. Compared with insect expression, *Escherichia coli (E.coli)* is an efficient prokaryotic expression system as many significant benefits in terms of low cost, ease-of-use and scale preparation.

Generally, immunogenic protein with high-molecular-weight can induce stronger immune response than low-molecular-weight protein [15]. Calreticulin (CRT) is a highly conserved endoplasmic reticulum luminal Ca^2+^-binding protein and found to be involved in cellular processes (e.g., calcium storage and chaperone function) [16]. Numerous studies primarily focused on its roles in protein folding and polymerization [17, 18]. Recombinant truncated CRT in polymers, as compared with monomers, can induce higher level of immune response [18]. Furthermore, CRT fused foreign proteins also formed into polymers and showed excellent immunogenicity of the foreign proteins [19]. In the present study, high-yield Cap-CRT fusion protein was expressed in *E.coli*, and the recombinant protein, rP5F could form into immunogenic polymers. Mice immunized with rP5F efficiently mounted humoral and cellular immune responses, and decrease the infection rate against PCV2-challenge, suggesting that rF5P could be a potential subunit vaccine candidate.

## Result

### Expression of cap-CRT fusion proteins and purification of rF5P

**Fig. 1:**
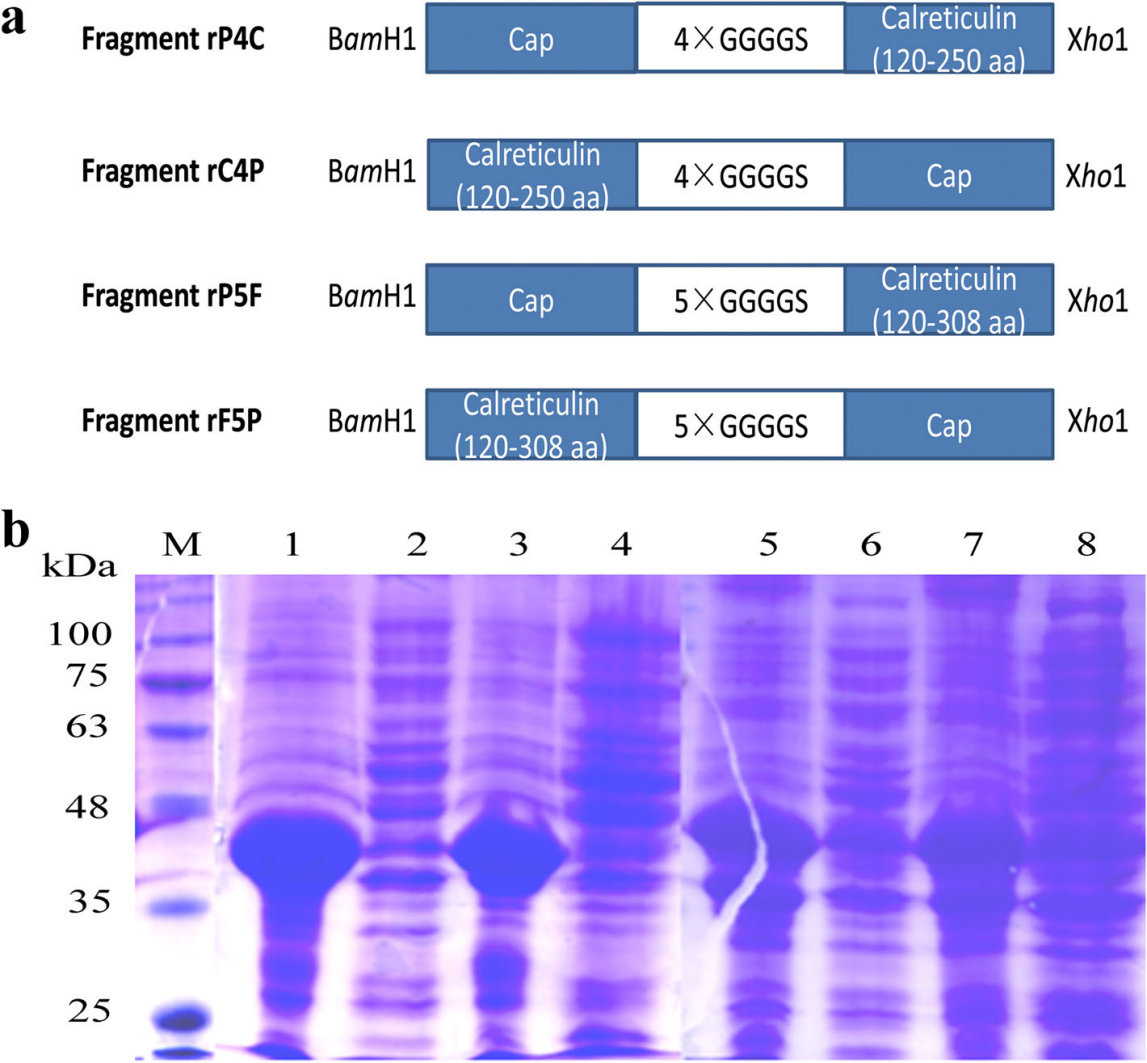
The schematic structure (**a**) and SDS-PAGE (**b**) of four recombinant Cap-CRT fusion proteins. **a** Fragments were used for constructing the recombinant proteins. Blue squares represent completely *cap* of PCV2 and truncated calreticulin. GGGGS in grey are linkers between *cap* and calreticulin. Each fragment is encoded by B*am*HI and X*ho*I, respectively. **b** Solubility of rP4C, rC4P, rP5F and rF5P induced by IPTG at 37 °C. M: protein ladder; Lane 1,3,5,7: precipitate of pET-28a-rP4C/rC4P/rV5P/rF5P; Lane 2,4,6,8: supernatant of pET-28a-rP4C/rC4P/rV5P/rF5P

**Fig. 2:**
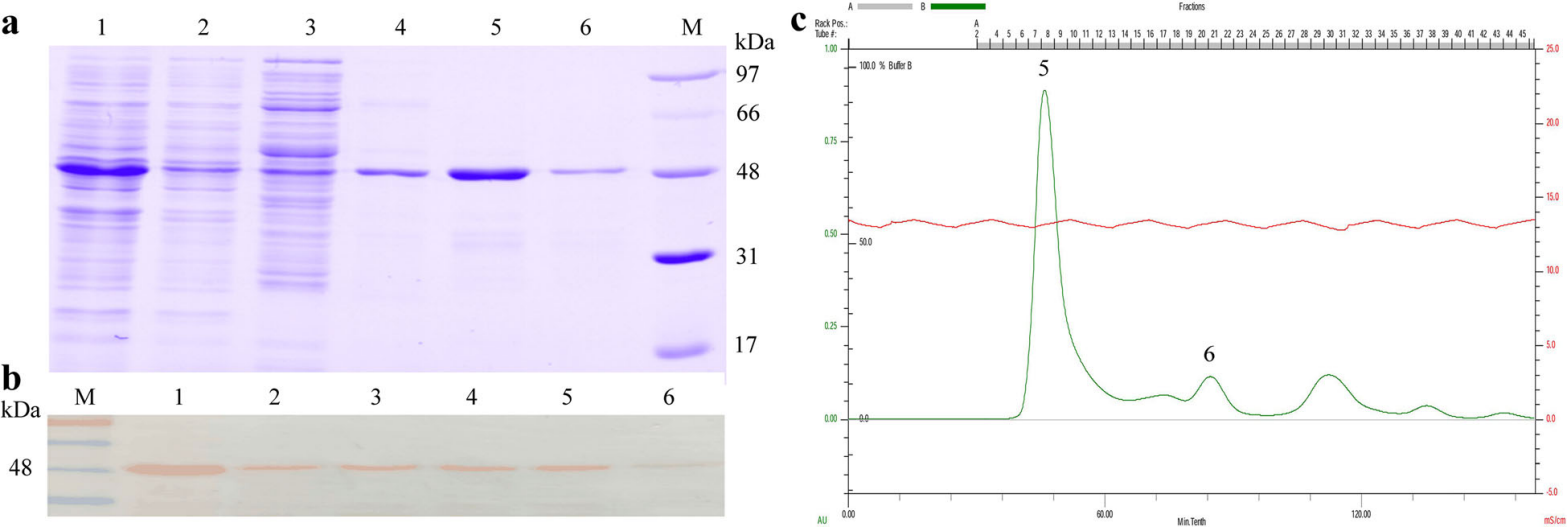
Purification and identification of rF5P. SDS-PAGE (**a**), Western-Blot (**b**) and Size-exclusion chromatography (**c**) of rF5P. M: protein ladder; Lane 1: lysate of rF5P; Lane 2: supernatant after settling the Ni-NTA resin by gravity; Lane 3: supernatant after washing resin; Lane 4: fraction after eluting (purified rF5P); Lane 5: the first peak of flow through by Superdex 200 pg (enriched rF5P); Lane 6: the third peak

The Cap-CRT fusion proteins (rP4C, rC4P, rP5F and rF5P) were successfully expressed in *E. coli*, whereas all of them led to inclusion bodies (IBs) at 37 °C (Fig. 1b) (Supplementary Material Original Fig. 1b). SDS-PAGE indicated that only rF5P achieving soluble expression with a molecular mass of 48 kDa at low temperature (25 °C) for 16 h (Fig. 2a lane 1). After rF5P was purified by Ni-NTA affinity chromatography, its quality was nearly 0.5 mg/mL with a purity of about 90% (Fig. 2a lane 4) (Supplementary Material Original Fig. 2a).

The purified rF5P by Ni-NTA was eluted from the Superdex 200 pg (26/60) gel filtration column. The target protein was presented as the first and highest peak, which beyond the detection limit of the column, suggesting that rF5P could form high-molecular-weight polymers (Fig. 2c lane 5). Besides, enrichment was also detected after elution from the column as the quality of rF5P was about 0.65 mg/mL (Fig. 2a, c lane 5). The results of Western Blot suggested that rF5P reacted specifically with anti-His mAbs (Fig. 2b) (Supplementary Material Original Fig. 2b). The third peak also recognized anti-His mAbs, revealing that only a small fraction of rF5P might exist in the form of monomer (Fig. 2b, c lane 6).

### Characterization of rF5P

**Fig. 3:**
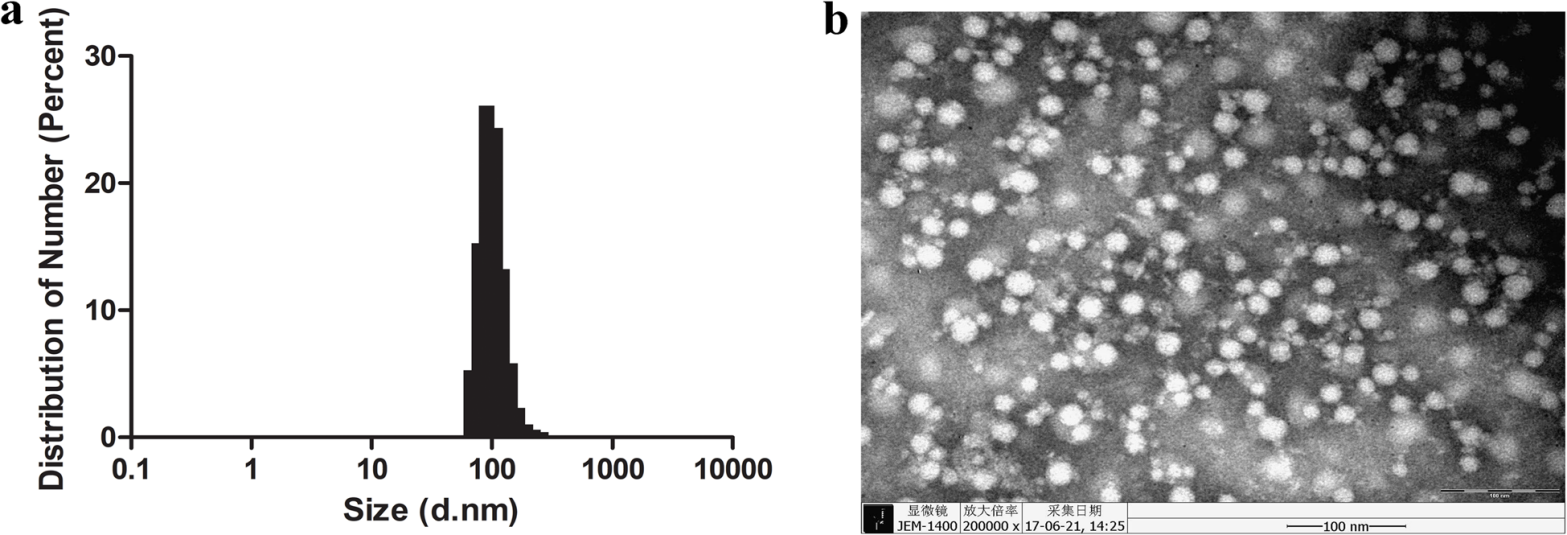
Characterization of rF5P. **a** Negative staining electron microscopy of rF5P, bar size, 100 nm. **b** Dynamic light scattering result of rF5P

To examine the morphology of high-molecular-weight polymers, the purified rF5P was analyzed under a TEM. The observed result revealed that rF5P was assembled into a spheroidal particle with a diameter of 30 nm, whereas the size distribution of the particles was not exactly the same, as shown in Fig. 3a, suggesting that there might be some incompletely assembled protein fragments. The DLS result indicated that the average hydrodynamic diameter of rF5P was about 100 nm (Fig. 3b). The sizes of rF5P particles observed using the two methods were not consistent, probably attributed to the hydration radius detected by DLS was larger than the theoretical or real value.

**Fig. 4:**
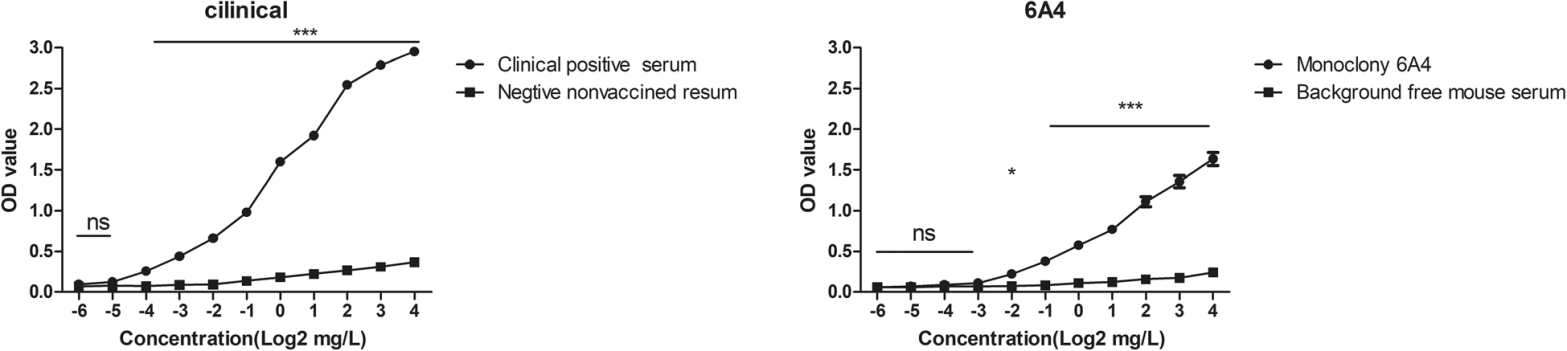
Antigenic characterizations of rF5P using swine clinical positive sera (**a**), anti-PCV2 mAbs 6A4 (**b**) and anti-His mAbs (**c**) by ELISA, and the results are expressed as mean OD value ± SEM, the statistical significance differences between each group was analyzed by two-way ANOVA statistical analysis, *P < 0.05, **P < 0.01, ***P < 0.001, ns represented not significant

The results of the antigenic analysis showed that rF5P could recognize clinical positive serum and anti-PCV2 mAbs 6A4, which indicated that rF5P had the similar characters as intact particle (Fig. 4). Compared with clinical positive serum, the mAbs 6A4 showed a relative weaker ability to recognize rF5P (Fig. 4b). However, the rF5P exhibited a high background interference of clinical negative serum, which probably associated with the complexity of the field sample (Fig. 4a).

### PCV2-specific humoral immune response

**Fig. 5:**
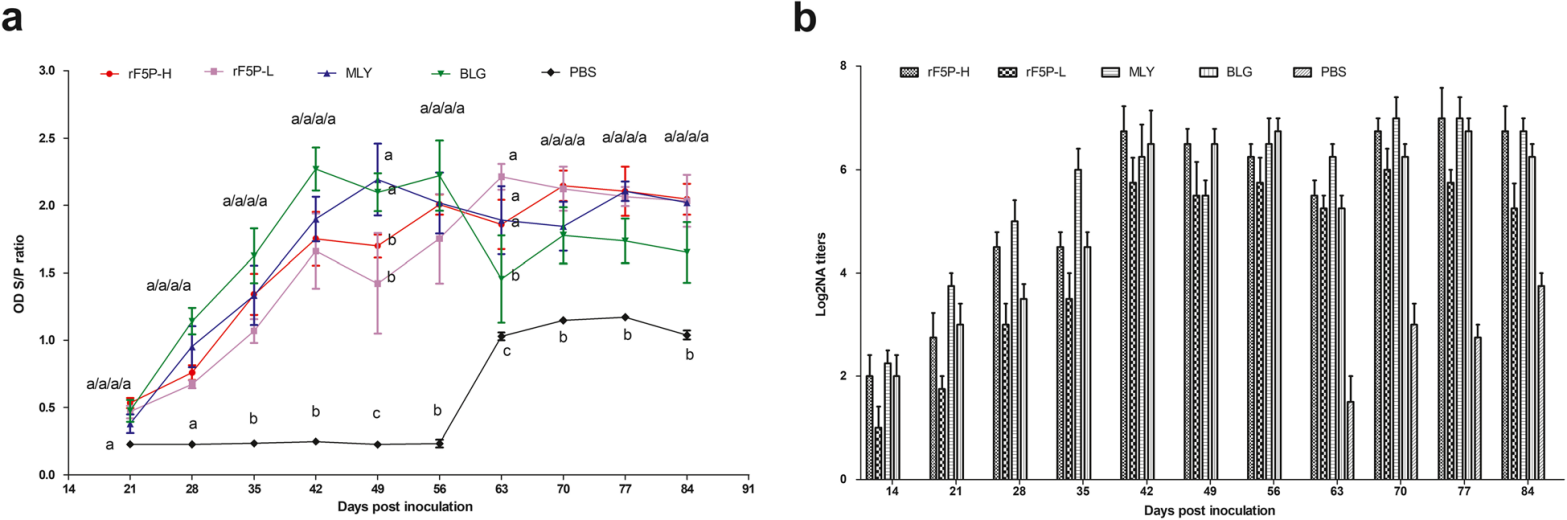
Detection of PCV2-specific immune responses in mice. Groups of mice (*n* = 6) were immunized with 30 μg and 15 μg of rF5P, commercial inactivated Circovac® vaccine (Merial), subunit vaccine Ingelvac CircoFLEX® (Boehringer Ingelheim) and PBS in injection, Blood samples were collected for PCV2-speicific IgG titers(**a**) and virus neutralization antibody (**b**). Titers of antibodies are expressed as mean ± SEM. Different letters (**a**, **b**, and **c**) indicate statistically significant difference (*P* < 0.05) among groups

Indirect ELISA was performed to evaluate PCV2-specific humoral immune response induced by rF5P in mice. Figure 5a shows that compared with the PBS group, PCV2-specific antibodies appeared at 21 dpi in all groups and increased with the advancement of the immune process. The antibody levels of MLY and BLG groups were overall higher than those of rF5PH and rF5PL groups before virus challenging, but the contrary phenomenon happened after that. During the entire immune process, the levels of rF5PH group were higher than those of the rF5PL group, whereas there was no significant difference between them. No antibody was produced in the PBS group before the challenge, and the antibody level increased immediately at 7 days after challenge and reached peak at 14 days.

Whether the antibodies generated by immunized mice could neutralize the virus, NA was adopted to further detect the PCV2-specific humoral immune response. The results indicated that all immune groups produced neutralizing antibodies except the PBS group, which were consistent with the results of indirect ELISA. The NA titers of rF5PH groups were higher compared with those of MLY and BLG at 42 and 49 dpi (Fig. 5b). After the challenge, NA titers in the PBS group increased rapidly and reached 1:16 at 4 weeks. Besides, the NA level in other immune groups decreased at 63 dpi (1 week after challenge); it returned to the level of pre-challenge at 70 dpi and remained unchanged until the completion of the test.

### Lymphocyte proliferative response and cytokine assay

**Fig. 6:**
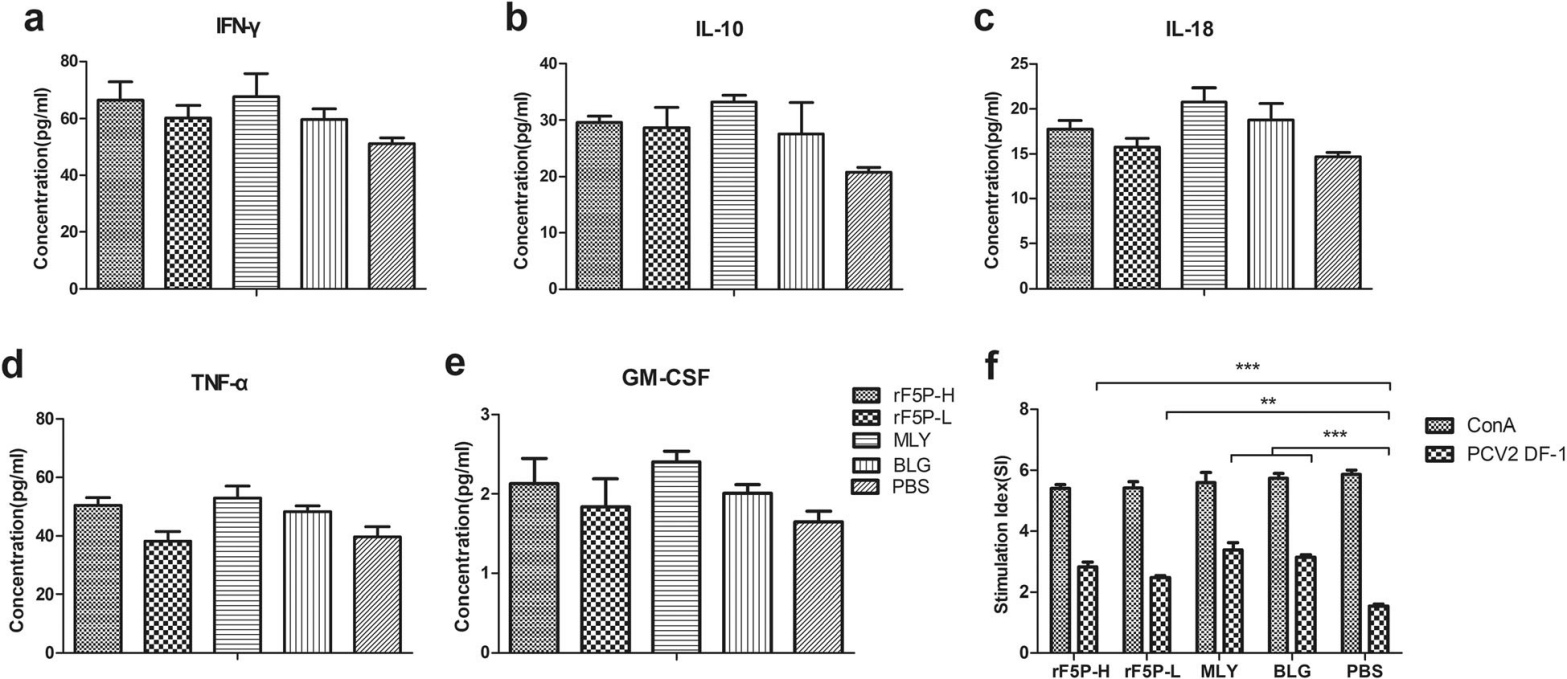
Analysis of cytokines secreted by lymphocyte of mice. Lymphocytes isolated from the spleen of mice at 56 dpi were stimulated with PCV2 strain DF-1 for 72 h, the supernatants were collected to detect the concentrations of cytokine of IFN-γ (**a**), IL-10 (**b**), IL-18 (**c**), TNF-ɑ (**d**), GM-CSF (**e**) by ELISA method and the T-lymphocyte proliferation (**f**) . Date are shown as mean ± SEM, statistical differences between each group was measured by one-way ANOVA, *P < 0.05, **P < 0.01, ***P < 0.001, ns represented not significant

Three mice in each group were sacrificed to isolate lymphocyte for lymphocyte proliferation and cytokine quantification through PCV2 strain DF-1 stimulation. The lymphocyte proliferative responses were detected in all immunized groups aside from the mock group. The SIs of rF5PH, MLY and BLG groups were significantly higher than that of the PBS group (*P* < 0.01), and there was no significance between the four immunized groups (*P* > 0.05) (Fig. 6f). The results suggested that cytokine levels were slightly higher in all the immune groups than the mock group, whereas there was no regular correlations and significant difference in the values (Fig. 6a-e).

### Quantification of PCV2 in tissues

**Fig. 7:**
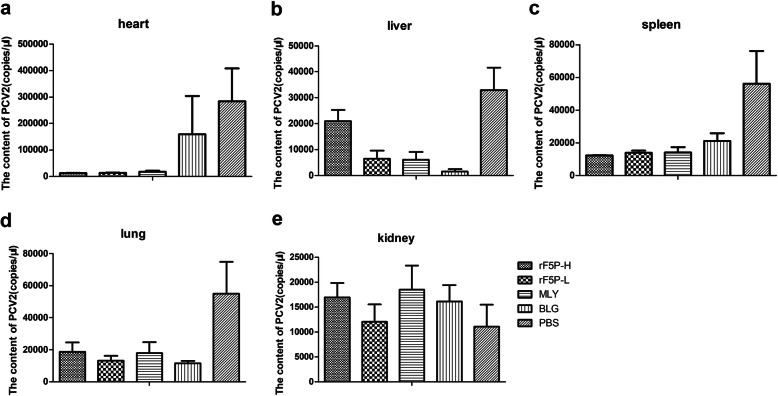
Protection from PCV2 strain DF-1 challenge in mice. All of the mice were challenged with 100 μL of 10^6.5^ TCID50/mL of the PCV2 strain DF-1 at 56 dpi and examined for 28 days. Spleens were isolated and the genomes were extracted to measure the content of PCV2 using quantitative real-time PCR. Date are shown as mean ± SEM, statistical differences between each group was measured by one-way ANOVA, *P < 0.05, **P < 0.01, ***P < 0.001, ns represented not significant

PCV2 DNA extracted from different tissues of all experimental groups post-challenge was quantified using real-time fluorescent quantitative PCR. Figure 7 suggested that excepted kidneys, the PBS group showed a significantly higher viral load than the other groups. The amounts of virus in the spleens and lungs of the immunized groups were lower than that in the PBS group (*P* < 0.05), and it showed no difference between the immunized groups (Fig. 7c, d). The rF5P groups exhibited the highest viral loads in the livers (Fig. 7b), but the lowest in the hearts (Fig. 7a). There was no difference among all groups in the kidneys (Fig. 7e). All the results revealed that mice immunized with rF5P could effectively reduce viral loads in organs against the PCV2 challenge.

## Discussion

PCV2, an agent of PCVDs, acts as a vital economical viral pathogen affecting the global swine industry. Vaccination has been demonstrated as a feasible means to control PCVAD. In this study, the Cap-CRT fusion proteins which could form into high immunogenic polymers were first produced in *E. coli*. Though there are multiple protein expression systems for protein expression in vitro, each system exhibits features and advantages, it also has limitations such as low yield and high cost which hinder the development of the recombinant protein into a truly useful vaccine. Meanwhile, *E. coli* prokaryotic expression system has been extensively adopted for recombinant protein production in laboratories and industry for its simplicity, rapid growth rate and relatively low cost.

Studies on Cap proteins focus on their abilities to self-assemble into virus-like particles (VLPs) and thus exert immune effects as an entire virus, which also prove that large molecular particles have stronger immune effects than monomer proteins [20]. However, the expression of recombinant proteins in *E.coli* often results in insoluble and/or nonfunctional IBs which may due to rapid synthesis and lack of post translational modification. CRT has been shown to be able to self-assemble effectively and acts as a chaperone to help dissolve and form the correct structure [21]. Three fourths design of Cap-CRT fusion proteins formed into IBs, only the rF5P transformed into soluble macromolecular particles in vitro by optimizing the expression conditions. However, the observations of TEM and DLS revealed that the particle radius was not the same, probably attributed to the DLS of hydrated radius larger than the theoretical or actual size. Besides, compared with other VLPs reports, the rF5P did not form VLPs.

The BALB/c mouse is one of the animal models, as it has a clear background and frees from external interference, it is the most extensively used in PCV2 inactivated or subunit vaccine researches [9, 10]. Though mice may not be an ideal animal model to resemble PCV2 infection as observed for pigs, PCV2 can infect and replicate in some mouse strains including BALB/c mouse when used with the appropriate inoculating dose and administered route. In the present study, the BALB/c mouse model was used to assess the immunogenicity and protective capabilities of an experimental vaccine based on the recombinant Cap-CRT fusion protein expressed in *E.coli*.

The Cap-CRT fusion protein (rF5P) induced production of PCV2-specific ELISA antibodies and neutralization antibody against PCV2 were detected, the PCV2-specific ELISA antibodies were positively correlated with the neutralization antibodies, which was consistent as described in Zhu [22]. The specific antibody levels of protein groups were lower than those of commercial vaccine groups before virus challenge, but it went opposite after the virus challenge. The neutralizing antibody levels of protein groups were slightly lower than commercial vaccine groups during the immune process. Both the protein and the commercial vaccine groups induced only part of the cellular immune response. Under the stimulation of PCV2, T lymphocytes proliferated significantly, whereas various cytokines were irregularly secreted. PCV2 infections mainly induce fetal and neonatal mortality, and the level of viruses in the tissues of PCV2 infected mice is a good indicator of the antiviral effects of any vaccine, and it primarily occurred in the lymphoid tissue and spleen [23]. After the challenge test, the viral loads in the spleens of mice in the protein and commercial inactivated vaccine group were significantly lower than those in the mock group. It was also slightly effective in the organs of the heart, liver and lung, which was not completely consistent with Wang’s research, PCV2 mainly deposited in the lungs [9]. The humoral immune response showed no significant difference between protein groups and commercial vaccine groups. Overall, the humoral and cellular immune levels of rF5P groups were similar to the two types of commercial vaccine groups, and the aggregate performance of rF5P was closer to BLG subunit vaccine. Our results clearly verified that the Cap-CRT fusion protein (rF5P) elicited humoral and part of cell mediated immune responses comparable to commercial inactivated and subunit vaccines, and protected mice against epidemic PCV2 strain DF-1 challenge.

## Conclusions

To sum up, this paper first describes that the PCV2 Cap protein fused with truncated calreticulin (rF5P) could be soluble expressed into immunogenically polymers in *E. coli*. Vaccination of mice elicited humoral and part of cellular immune responses comparable to the commercial inactivated and subunit vaccines, and significantly reduced the viral loads in tissues subsequent to a viral challenge. Besides, the immune effect of Cap-CRT fusion protein requires further verifications in pigs as the natural hosts of PCV2. The rF5P can potentially develop a subunit vaccine against PCV2 infection.

## Methods

### Cells and virus

PK-15 cells (ATCC™ CCL-33) were cultured in Dulbecco’s Modified Eagle Medium (DMEM; Gibco) containing 10% fetal bovine serum (FBS, HyClone), 100 IU/mL Penicillin and 100 mg/L Streptomycin (InvivoGen, France) at 37 °C in a 5% CO_2_ atmosphere. PCV2 strain DF-1 (GenBank Accession Number: JN119255) was grown in PK-15 cells and utilized for virus neutralization assay (NA) and experimental challenge.

### Experimental animals

Thirty female BALB/c mice of 4 weeks old weighing 14-18 g were chosen arbitrarily and purchased from the Experimental Animal Center of Zhengzhou University. The experimental mice were randomly separated into five groups and given 5 days to acclimate the housing environmental conditions (temperature: 22 ± 3 °C, humidity: 55 ± 15%, lighting: 12 h light/dark cycle). The mice were allowed free access to clean water and food. The animal experiments were carried out according to the Animal Experiment Committee of Henan Academy of Agricultural Sciences (Approval number SYXK 2014–0007). All animals received humane care in compliance with good animal practice according to the animal ethics procedures and guidelines of China. All sections of this report adhere to the ARRIVE Guidelines for reporting animal research [24].

### Plasmid construction

As shown in Fig. 1a, complete *Cap* gene of PCV2 (GenBank Accession No. AY686763) was fused with the truncated calreticulin (120–308 aa/120–250 aa) (GenBank Accession No. EU639407) at N/C terminal using 4 × GGGGS or 5 × GGGGS linker. All these four recombinant fragments, named rP4C/rC4P/rP5F/rF5P, were synthesized after codon optimization by Genscript. All the plasmids were inserted into pET-28a in B*am*HI and X*ho*I sites and then transformed into *E. coli* BL21 (DE3) competent cells, respectively.

### Protein expression and purification

All the positive clones were cultured in Luria-Bertani (LB) medium containing 50 mg/L kanamycin and induced for protein expression with 0.1 mM IPTG at 37 °C for 6 h. The parameters of protein expression were optimized according to IPTG concentrations (0.1 mM, 0.2 mM), induction temperature and time (18 °C for 24 h, 25 °C for 16 h). Protein expression was verified by sodium dodecyl sulfate polyacrylamide gel electrophoresis (SDS-PAGE). The optimal harvest cells were suspended in lysis buffer (50 mM PB, 150 mM NaCl, 5% (*w/v*) Glycerol, 5% (*w/v*) Triton X-100, 2 mM EDTA, 2 mM DTT, pH 7.0) and then lysed by sonication (99 cycles of 2 s On/5 s Off, amp 25%)). After centrifugation, the precipitation was removed and the supernatant of rF5P was purified by Ni-NTA affinity chromatography. After washing the Ni-NTA column (Invitrogen, USA) with wash buffer (50 mM PB, 150 mM NaCl, 30 mM imidazole, pH 7.0), rF5P was eluted with elution buffer (50 mM PB, 150 mM NaCl, 250 mM imidazole, pH 7.0). Protein fractions were analyzed by SDS-PAGE.

The purified rF5P was enriched and analyzed by size-exclusion chromatography with Superdex 200 prep grade (pg) (26/60) gel filtration column (GE Healthcare, USA). The samples were eluted using lysis buffer at a flow rate of 1 mL/min and detected at 280 nm wavelength. The collected fractions were identified by SDS-PAGE and Western Blot and then quantified using BCA Protein Assay Kit (TIANGEN, China).

### Characterization of rF5P

The purified rF5P was observed under a transmission electron microscopy (TEM) using the negative staining method and dynamic light scattering (DLS) according to the previous study [25].

### Antigenicity analysis of rF5P

Indirect enzyme-linked immunosorbent assay (ELISA) was performed to test the antigenicity of rF5P with swine clinical positive/negative serum and mouse anti-PCV2 monoclonal antibodies (mAbs) 6A4 (Abcam, USA). The ELISA procedure was operated as routine.

### Vaccination and challenge in mice

Thirty female BALB/c mice were randomly divided into 5 groups (*n* = 6). The mice were inoculated subcutaneously with 30 μg and 15 μg of rF5P as Group rF5PH and Group rF5PL, respectively; 50 μL of commercial inactivated Circovac® vaccine (Merial), subunit vaccine Ingelvac CircoFLEX® (Boehringer Ingelheim) and PBS were classified as positive and negative controls, named as Group MLY, BLG and PBS, respectively.

The rF5P was diluted in 50 μL of PBS and then emulsified with 50 μL of Complete Freund’s adjuvant for the first immunization, and subsequently with 50 μL of Incomplete Freund’s adjuvant for booster at an interval of 4 weeks. At 56 days after the first immunization, 3 mice from each group were sacrificed for both lymphocyte proliferation assay and cytokine production. In order to reduce the pain of mice to the greatest extent, cervical dislocation was chosen to kill them. It is the fastest method to make the spinal cord and brain spinal cord disconnected, so that the central nervous system instantly lost control of the whole body, which is in line with the requirements of animal welfare. The rest alive mice received 100 μL of 10^6.5^ (TCID_50_)/mL PCV2 strain DF-1, and they were monitored for the following 28 days. Next, the mice were sacrificed for PCV2 content in different organs. Blood samples were collected from the tail veins each week.

### Antibody response in mice

The serum samples taken at each point post immunization were monitored for specific antibodies using Porcine circovirus type 2 ELISA antibody test kit (KeQian, China). Operation steps followed the manufacturer’s instructions.

### Neutralization assay

The abilities of all serum samples to neutralize the PCV2 strain DF-1 were assessed using virus NA. In brief, 50 μL sera pre-treated at 56 °C for 30 min were diluted in a serial two-fold way from 1:2 to 1:1024 and mixed with an equal volume of virus (400 TCID_50_) at 37 °C for 1 h. The serum-virus complex was transferred into confluent PK-15 cells in each well and then incubated at 37 °C for 72 h. Since no visible cytopathic effect was verified, immunoperoxidase monolayer assay (IPMA) was performed to ascertain the presence of the virus [10]. Virus neutralization titer was expressed as the highest dilution as log_2_NA in which no higher than 80% reduction of virus replication was detected as compared with the virus control.

### Spleen lymphocyte proliferation assay

Spleens of mice from each group were removed at 56 days post inoculation (dpi). The spleen lymphocytes were isolated by Lydroxypropylmethyl Cellulose (Solarbio, China) and then resuspended in RPMI 1640 medium containing 10% FBS. Lymphocyte proliferation assay was performed by cell counting kit-8 assay (Beyotime Biotechnology, China) as previously described [26]. T lymphocyte proliferation was represented as the stimulation index (SI), the ratio of the mean reading of stimulated wells to unstimulated ones.

### Analysis of cytokine production by activated lymphocytes

The supernatants from the spleen lymphocytes employed in the proliferation assay were removed and adopted to analyze cytokines. The assays were performed using commercially available mice IFN-γ, IL-10, IL-18, TNF-ɑ and GM-CSF ELISA kits (USCN Life Science, China) following the manufacturer’s instructions.

### Determination of PCV2 in tissue

PCV2 DNA from different organs (heart, liver, spleen, lung and kidney) of all groups at 28 days post-challenge was quantified by real-time fluorescent quantitative PCR as previously described [27]. The viral load was calculated according to the standard curve plotting Ct values against different dilutions of a standard plasmid.

### Statistical analyses

GraphPad Prism version 5.00 (USA) analysis of variance (ANOVA) was performed. The data is expressed as the mean ± SEM. Statistical significance was found by two-way or one-way ANOVA at*P < 0.05, **P < 0.01, ***P < 0.001; ns represents no statistical significance. All the experimenters were not blinded to any stage of the experiment.

## Supplementary information

**Additional file 1.**

**Additional file 2.**

**Additional file 3.**

## Data Availability

The datasets used and/or analyzed during the current study are available from the corresponding author on reasonable request.

## Abbreviations

PCV2: Porcine circovirus type 2
PCVDs: Porcine circovirus diseases
PCVAD: Porcine circovirus associated diseases
Cap: Capsid protein
ORFs: Major open reading frames
CRT: Calreticulin
*E.coli*: *Escherichia coli*
IBs: Inclusion bodies
SDS-PAGE: Sodium dodecyl sulfate-polyacrylamide gel electrophoresis
ELISA: Indirect enzyme-linked immunosorbent assay
DLS: Dynamic light scattering
TEM: Transmission electron microscopy
MAbs: Monoclonal antibodies
IPMA: Immunoperoxidase monolayer assay
NA: Neutralization assay
Dpi: Days post inoculation
SI: Stimulation index
RT-PCR: Real-time PCR
IL: Interleukin
TNF: Tumor necrosis factor
IFN: Interferon
GM-CSF: Granulocyte-macrophage colony stimulating factor
