# Supplementary material for: PCV cap proteins fused with calreticulin expressed into polymers in Escherichia coli with high immunogenicity in mice
Source: BMC Vet Res. 2020 Aug 27;16:313. doi: 10.1186/s12917-020-02527-9 (PMC7450944; doi:10.1186/s12917-020-02527-9)
Supplement: Supplementary file 1 — Additional file 1. [file 12917_2020_2527_MOESM1_ESM.docx]

**Figure 1b original:**


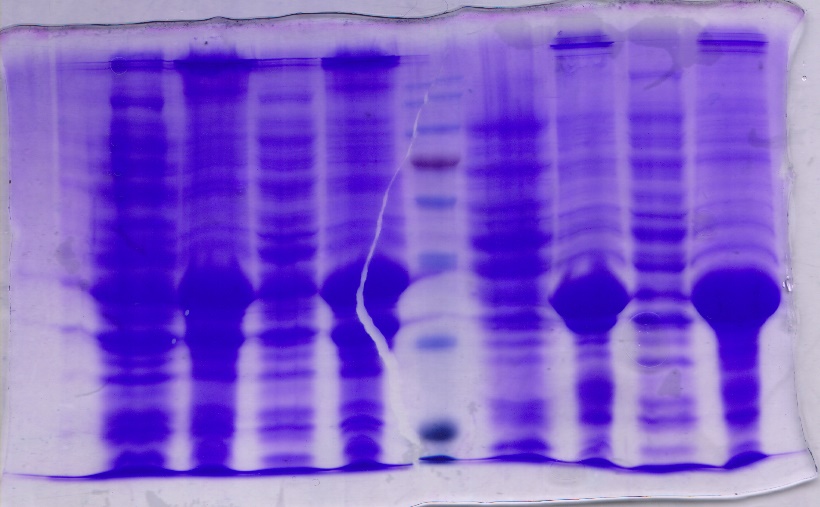


**Figure 1b** was provided with full-length gel images as additional file, and figure legends was added. Since the marker in the original image is in the middle of the gel, the lane order is adjusted.


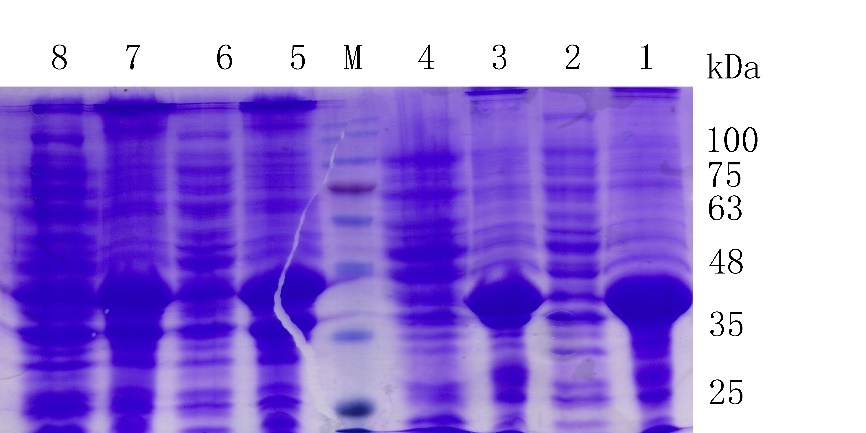


**Fig. 1.** The SDS-PAGE (b) of four recombinant Cap-CRT fusion proteins. (b) Solubility of rP4C, rC4P, rP5F and rF5P induced by IPTG at 37 ℃. M: protein ladder; Lane 1,3,5,7: precipitate of pET-28a-rP4C/rC4P/rV5P/rF5P; Lane 2,4,6,8: supernatant of pET-28a-rP4C/rC4P/rV5P/rF5P.
