# Supplementary material for: PCV cap proteins fused with calreticulin expressed into polymers in Escherichia coli with high immunogenicity in mice
Source: BMC Vet Res. 2020 Aug 27;16:313. doi: 10.1186/s12917-020-02527-9 (PMC7450944; doi:10.1186/s12917-020-02527-9)
Supplement: Supplementary file 2 — Additional file 2. [file 12917_2020_2527_MOESM2_ESM.docx]

**Figure 2a original:**





**Figure 2a** was provided with full-length gel images as additional file, and figure legends was added. Since the original image of the gel is mirror flip, the lane order is adjusted. Lane m and 7-8 are cropped as useless in this test.


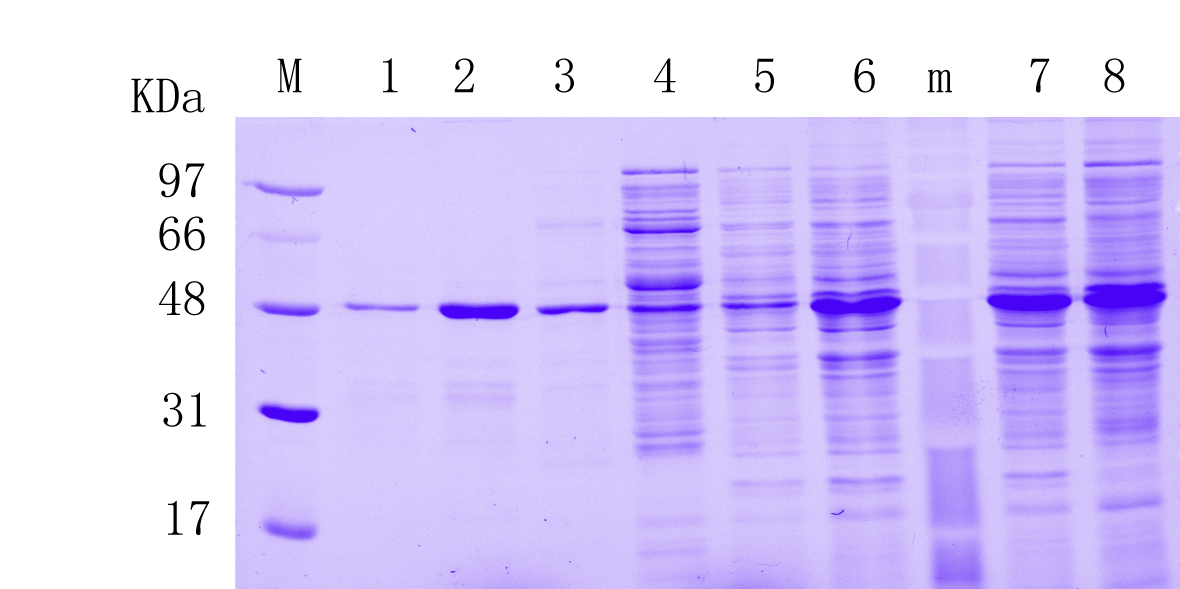


**Fig. 2.** Purification and identification of rF5P. SDS-PAGE (a) of rF5P. M: protein ladder; Lane 1: the third peak; Lane 2: the first peak of flow through by Superdex 200 pg (enriched rF5P); Lane 3: fraction after eluting (purified rF5P); Lane 4: supernatant after washing resin; Lane 5: supernatant after settling the Ni-NTA resin by gravity; Lane 6: lysate of rF5P; m: fluorescence protein ladder; Lane 7: lysate of rF5P; Lane 8: precipitate of rF5P.
