# Supplementary material for: PCV cap proteins fused with calreticulin expressed into polymers in Escherichia coli with high immunogenicity in mice
Source: BMC Vet Res. 2020 Aug 27;16:313. doi: 10.1186/s12917-020-02527-9 (PMC7450944; doi:10.1186/s12917-020-02527-9)
Supplement: Supplementary file 3 — Additional file 3. [file 12917_2020_2527_MOESM3_ESM.docx]

**Figure 2b original**:


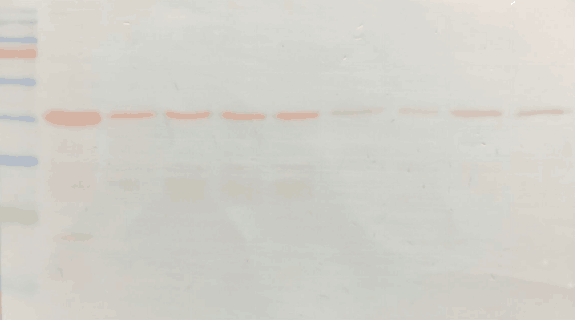


**Figure 2b** was provided with full-length gel images as additional file, and figure legends was added. Lane 7-9 and redundant margens are cropped as useless in this test.


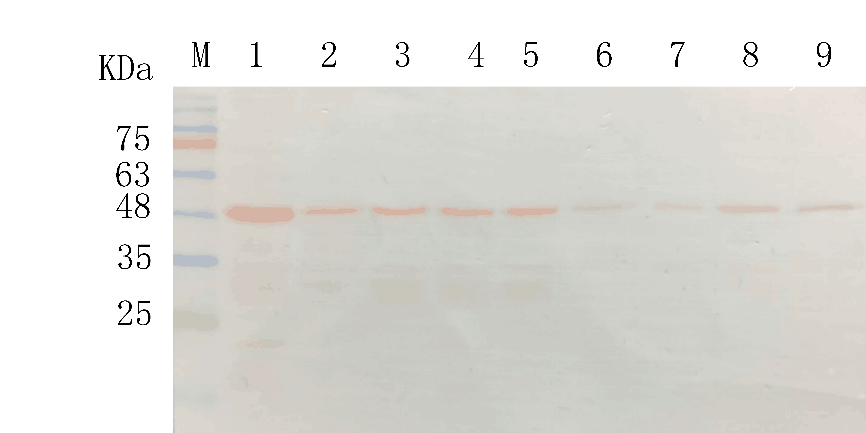


**Fig. 2.** Purification and identification of rF5P. Western-Blot (b) of rF5P. M: protein ladder; Lane 1: lysate of rF5P; Lane 2: supernatant after settling the Ni-NTA resin by gravity; Lane 3: supernatant after washing resin; Lane 4: fraction after eluting (purified rF5P); Lane 5: the first peak of flow through by Superdex 200 pg (enriched rF5P); Lane 6-7: the third peak; Lane 8-9: the third peak after ultracentrifugation.
